# Effects of Mental Simulation of Future Waterpipe Tobacco Smoking on Attitudes, Perceived Harms and Intended Use among Young Adults

**DOI:** 10.1007/s10865-021-00245-7

**Published:** 2021-08-18

**Authors:** Isaac M. Lipkus, Darren Mays, Paschal Sheeran, Wei Pan, Linda D Cameron, Felipe De Brigard

**Affiliations:** Duke University School of Nursing; Center for Tobacco Research, The Ohio State University Comprehensive Cancer Center, Department of Internal Medicine, The Ohio State University College of Medicine, Columbus, USA; University of North Carolina, Chapel Hill; Duke University School of Nursing; University of California, Merced; Duke University

**Keywords:** Waterpipe tobacco smoking, mental simulations, attitudes

## Abstract

The desire to engage in waterpipe tobacco smoking (WTS) may occur when smokers and nonsmokers conjure positive mental simulations of WTS. However, effects of these simulations on desire to smoke waterpipe tobacco and potential mediators are unexplored. This research addressed these effects among young adult waterpipe tobacco smokers and nonsmokers. Two online studies were conducted with adults ages 18–30. In Study 1, 200 smokers, 190 susceptible nonsmokers, and 182 nonsusceptible nonsmokers were randomized to mentally simulate or not WTS in the future. In Study 2, 234 smokers and 241 susceptible nonsmokers were randomized to four arms: no simulation or simulations that varied valence of experience (positive, negative or no valence provided). Main outcomes were immediate desire to smoke waterpipe tobacco, cognitive and affective attitudes, and perceived harms. In Study 1, mental simulations increased the desire to smoke waterpipe tobacco among smokers. In Study 2, asking participants to simulate WTS positively or with no valence instruction increased desire to smoke relative to negative valence instruction or no simulation. Negative simulations reduced perceived probability of smoking within a month compared to positive simulations. Effects on desire to engage in WTS were mediated by cognitive and affective attitudes among susceptible nonsmokers and by cognitive attitudes among smokers. These findings suggest that exploring when and how often mental simulations about WTS are evoked and their potency for promoting prevention and cessation of WTS merit further attention.

## Introduction

In the US, waterpipe tobacco smoking (WTS) is a public health concern due to WTS-related health risks such as poorer pulmonary function, heart disease, and cancer (Akl et al., 2010; Mamtani et al., 2017; Montazeri et al., 2017; Raad et al., 2011; Rezk-Hanna & Benowitz, 2018; Waziry et al., 2017). Many psychological (e.g., positive attitudes, low perceived harms), social (e.g., norms, friends/family influence), cultural, and marketing (e.g., enticing advertisements, limited health warnings) influences have contributed to WTS appeal and spread, especially among young adults (Akl et al., 2015; Lopez et al., 2017). Nationally in 2017, about 7% of 18–24 year olds engaged in WTS during the last 30 days (United States Department of Health and Human Services, National Institutes of Health, National Institute on Drug Abuse, United States Department of Health and Human Services, Food and Drug Administration, Center for Tobacco Products, 2018); in some states, close to 19% of college educated 20–28 year olds smoked during the last 30 days (Loukas et al., 2019). In addition, many young adults who do not engage in WTS are open to trying it; that is, they are susceptible.Among college students, 27% to 51% are susceptible (Lipkus et al., 2015; Nuzzo et al., 2013; Roberts & Ferketich, 2020). In a nationally representative sample, 22% of young adults ages 18–30 were susceptible to WTS (Sidani et al., 2017). Of concern, susceptible individuals are more likely to initiate WTS (Lipkus et al., 2015; Sidani et al., 2017). Unfortunately, there is limited evidence for interventions to prevent WTS or enhance cessation in young adults (for reviews, see Jawad et al., 2016; Maziak et al., 2015; Sadeghi et al., 2019).

A factor that has not been explored in relation to WTS is smokers’ and nonsmokers’ mental simulations of engaging in future WTS. Humans spend considerable time contemplating the future and make plans accordingly to achieve desired and avoid undesired outcomes (Schacter et al., 2015; Taylor et al., 1998). These efforts often involve mentally simulating scenarios depicting possible future actions and corresponding outcomes. Episodic future thinking—that is, mental simulations of possible future events (Schacter et al., 2015)—has been shown to influence pro-social attitudes (Gaesser & Schacter, 2014), emotion regulation (Miloyan et al., 2016), and prospective memory (i.e., our ability to remember carrying out an intended action (Neroni et al., 2014), among other processes. Relatedly, and critical for the purpose of the current studies, episodic future thinking can influence decision-making, such as attenuating temporal discounting in decisions involving money (Peters & Büchel, 2010), and reducing calorie intake (Dassen et al., 2016), alcohol consumption (Snider et al., 2016), and cigarette smoking (Stein et al., 2016).

While it remains unclear how decisions to engage in WTS occur, mental simulations likely affect these decisions via deliberate and impulsive (i.e., experiential) processes. For example, deliberative processes that increase probability of use can include simulations of planned future waterpipe tobacco in enjoyable social situations, using appealing flavored tobacco, and when and where to smoke (e.g., a favored waterpipe tobacco establishment, home). Further, mental simulations of WTS are hypothesized to occur spontaneously by reflecting on past WTS experiences, environmental exposures about WTS, such as product advertisements and/or passing waterpipe tobacco establishments, as well as conversations the ensue on the topic in person or social media. In sum, mental simulations of WTS, triggered various ways, can increase the decision to engage in and use the product.

The extent to which imagery during episodic future thinking can influence intention to engage in WTS and potential mediators for this effect are unknown. For example,many young adults believe WTS is not harmful or addictive (Akl et al., 2015; Cornacchione et al., 2016; Hair et al., 2017; Heinz et al., 2013). These risk perceptions are associated with waterpipe use (Akl et al., 2015; Eissenberg et al., 2008; Hair et al., 2017; Primack et al., 2008; Sutfin et al., 2011; Villanti et al., 2015) and may be a potent reason why susceptible young adults experiment with WTS. Mental images about a health risk have been linked with risk perceptions as well as risk-related intentions and actions (Cameron, 2008; Slovic et al., 1991; Traczyk et al., 2015). In experimental studies, manipulations that induce mental images of harms of risky behaviors increase perceived risk (Lee et al., 2011; Sobkow et al., 2016) and reduce risk-related intentions and behaviors (Lee et al., 2011).

Mental simulations that lower perceived harms or create mental images involving positive experiences of future WTS may increase favorable cognitive and affective attitudes toward WTS (Mays et al., 2020). For example, according to the affect heuristic, lower perceived risks are associated with more positive attitudes toward the target of focus (Slovic et al., 2005). To the degree that mental simulations of future WTS entail positive thoughts, feelings, and sensory experiences (e.g., taste, smells), smokers and nonsmokers are expected to report lower perceived harms and more favorable cognitive (e.g., safe, useful) and affective (e.g., pleasant, satisfying) attitudes toward WTS (Trafimow & Sheeran, 1998). In turn, lower perceived risk and positive attitudes should correlate with a stronger desire to engage in WTS.

Mental simulations’ influence on attitudes, risk perceptions, and intentions is expected to be stronger among waterpipe tobacco smokers than among susceptible or nonsusceptible nonsmokers for the following reasons. According to the constructive episodic simulation hypothesis (Schacter & Addis, 2007), individuals use information stored in episodic memory to construct mental simulations of possible future events. When experienced events closely resemble those imagined, the clarity and vividness of the imagined event is stronger (De Brigard & Giovanello, 2012), and there is more overlap with neural regions engaged in episodic memory encoding and retrieval (Papies et al., 2017; Schacter et al., 2015). Given these processes, smokers’ mental simulations of WTS are expected to align with their assumed positive experiences of using the product and thus should promote stronger intentions and greater actual product use compared to WTS imagery in nonsmokers.

### Present research

In two online experimental studies, we examined how simulations influence perceived harms, attitudes toward, and desire to engage in WTS among waterpipe smokers and nonsmokers. The purpose of Study 1 was twofold. First, based on the constructive episodic simulation hypothesis, we examined as proof of concept whether smokers’ simulated experiences of WTS are more positive (e.g., more favorable thoughts, feelings, and physical sensations) and align more closely with their anticipated future experiences of WTS than susceptible and nonsusceptible nonsmokers. Second, we tested the following interaction hypotheses:

- Smokers and susceptible nonsmokers in the mental simulation arm will report more positive cognitive and affective attitudes as well as lower perceived harm of WTS compared to their counterparts in the control arm; nonsusceptible nonsmokers in the mental simulation arm will report more negative cognitive and affective attitudes as well as more harm about WTS compared to the control arm.
- Smokers and susceptible nonsmokers in the mental simulation arm will express a stronger desire to smoke waterpipe tobacco compared to their counterparts in the control arm; nonsusceptible nonsmokers in the mental simulation arm will report a lower desire to smoke waterpipe tobacco compared to participants in the control arm.

We examined further whether the positivity/negativity (i.e., valence) of simulated thoughts, feelings, and physical sensations correlated with the desire to smoke waterpipe tobacco and whether the association is mediated by perceived harms and attitudinal beliefs toward WTS. We expected that simulated future WTS that entailed more positive thoughts, feelings, and pleasant sensory experiences would be associated with a higher desire to smoke and these associations would be mediated by lower perceived harms and more favorable attitudes toward WTS. We expected these patterns to be moderated by smoking status such that they would hold more powerfully for smokers, followed by susceptible then nonsusceptible nonsmokers. In a second study we examined how the explicit manipulation of the valence of the simulation (e.g., positive or negative simulations) influenced attitudes toward, perceived risks and desire/intention to engage in WTS.

## Study 1: Mental Simulation and Desire to Smoke Waterpipe Tobacco

### Methods

#### Participants

Participants were recruited using the Internet crowdsourcing platform Amazon Mechanical Turk (AMT). Waterpipe tobacco smokers and (non)susceptible nonsmokers were recruited separately. Individuals first gave consent and then completed a screener to determine eligibility. To be eligible as a smoker, participants had to be 18 to 30 years of age, report WTS use within the last 30 days, and express not having quit. To be eligible as a (non)susceptible nonsmoker, participants had to be 18 to 30 years of age, report never having smoked waterpipe tobacco, not even a puff or two. Susceptibility status was based on a four-item scale: 1) “Do you think that you will smoke tobacco from a waterpipe soon?” 2) “Do you think that you will smoke tobacco from a waterpipe in the next year?” 3) “Do you think that in the future you might experiment with waterpipe tobacco smoking?” and 4) “If one of your best friends asked you to smoke tobacco from a waterpipe, would you?” (Lipkus et al., 2015). Response options included: “Definitely yes”; “Probably yes”; “Probably no”; and “Definitely no”. Participants were deemed susceptible to WTS if they responded other than “Definitely no” to one or more questions. Data quality assurance measures included prohibiting duplicate responses and using verification to prevent automated completion (i.e., by bots).

#### Mental Simulation Procedures

Using a between-subjects design, and ran as three separate studies by smoking and susceptibility status, smokers and nonsmokers were randomly assigned with equal probability to either a no WTS mental simulation control arm or to an experimental arm that asked participants to mentally simulate future WTS (Stein et al., 2016). The instructions for this task were “Imagine in vivid detail what it would be like for you to smoke waterpipe tobacco in the future. Let your mind roam free and fully immerse yourself in the experience of smoking waterpipe tobacco. What is going through your mind? What words and images describe your experience?” Participants were provided a box to write their responses. They were given as much time as needed to simulate WTS and respond. After this task, participants responded to the questions below, as did participants in the control arm. Participants who completed the survey were paid $2.00 to their AMT account.

#### Measures

Smokers and nonsmokers randomized to the mental simulation arm completed the following ratings to capture their simulated experience of future WTS.

*Valence of simulated experience* was captured by participants rating their overall thoughts, feelings, and physical sensations (e.g., taste, smell, touch, sounds) from 1= Very negative to 7=Very positive.

*Realism of simulated experience* was assessed by, “How realistic is this experience for you?” Response anchors were 1= Not at all realistic to 7=Very realistic.

*Likelihood of experiencing simulated event* was assessed by “How likely are you to experience smoking waterpipe tobacco in the future as you imagined it?” Response anchors were 1=No chance to 7=Certain to happen.

*Ease of simulating WTS* was captured by, “How hard or easy was it to imagine your experience of smoking waterpipe tobacco?” Response anchors were 1=Very easy to 7=Very hard.

*Effort simulating WTS* was assessed by, “How much effort did you put into imagining how it would be like for you to smoke waterpipe tobacco in the future?” Response anchors were 1= No effort at all to 7= A great deal of effort.

All participants provided demographic and tobacco smoking profile information as well as completing the assessments below.

##### Perceived harms of WTS.

For smokers, this was captured via four ratings, “What do you think is your chance of getting a serious smoking-related disease in your lifetime, such as cancer, lung disease, or heart disease, if you do not quit?” (1=No chance, 2=Very Unlikely, 3=Unlikely, 4=Moderate chance, 5= Likely, 6=Very likely and 7=Certain to happen); “What do you think is your chance of becoming addicted to nicotine in tobacco from waterpipe if you do not quit?” (same response options as above); “How worried are you about getting a serious smoking-related disease in your lifetime, such as cancer, lung disease, or heart disease, if you do not quit?” (1=Not at all to 7=Very); and “How worried are you about becoming addicted to nicotine in tobacco from waterpipe if you do not quit?” (1=Not at all to 7=Very). Among nonsmokers, the four questions were posed conditional on if they were to smoke and not quit (e.g., “What do you think is your chance of getting a serious smoking-related disease in your lifetime, such as cancer, lung disease, or heart disease, *if you were to smoke waterpipe tobacco and did not quit*?”). These four ratings, which all loaded on a single factor, were summed and averaged for smokers and nonsmokers (Cronbach’s alphas > .85).

##### Cognitive and affective attitudes about WTS.

Based on 9-point bipolar scales (Trafimow & Sheeran, 1998), five items captured cognitions (e.g., unsafe/safe, foolish/wise, useless/useful) and five items captured feelings (e.g., unpleasant/pleasant, revolting/gratifying, unsatisfying/satisfying) about WTS. The items for each subscale were summed and averaged (Cronbach’s alphas = .88 to .97). Pearson correlations between subscales for smokers and nonsmokers across the two conditions ranged from .52 to .80 (ps<.001).

##### Immediate desire to smoke.

All participants were asked, “How strong is your desire to smoke waterpipe tobacco right now?” Response anchors were 1=Not at all strong to 7=Very strong. We assessed desire because it should be a strong predictor of intention to engage in WTS (Perugini & Bagozzi, 2004).

#### Analytical methods

Mean differences in reactions to simulating WTS, perceived harm, attitudes, and desire to smoke WTS were tested using ANOVAs modelling the main effect of study arm, smoking status, and their interaction. Demographic variables, primarily age, sex, race, and education, did not modify the main pattern of effects and did not differ across study arms. Thus, we present the unadjusted means along with standard deviations and standard error of the mean. Analyses were two-tailed using *p*< .05 as statistical significance and conducted with SAS Version 9.4 (Cary, North Carolina). There were missing data for a few variables; however, overall missing was minimum (about 5.0% or less). Thus, the missing data were excluded analysis-by-analysis.

Among participants in the simulation arm, pathways from valence of thoughts, feelings, and physical sensations to desire to smoke, with perceived harm and attitudes as correlated mediators, were examined with smoking status as a moderator using multi-group structural equation models (SEM; Byrne, 2004). We first tested an unconstrained model that allowed mediational pathways to vary across smoking status. We then tested a constrained model that imposed invariance on the mediational pathways across smoking status. We used the nested chi-square (χ^2^) test statistic to compare the fit between the two models. An unconstrained model with better fit suggests that the mediation pathways vary by smoking status (i.e., strength of the pathways among the variables differ by smoking status). The model fit was assessed by commonly-used goodness-of-fit indices: chi-square (χ^2^), goodness of fit index (GFI), normed fit index (NFI), incremental fit index (IFI), relative fit index (RFI), comparative fit index (CFI), and root mean square error of approximation (RMSEA). Following recommended criteria for evaluating model fit indices (Hu & Bentler, 1999; Kline, 2015), a nonsignificant χ^2^ value (*p*> .05) indicates a good overall model fit to the data. For GFI, NFI, IFI, RFI, and CFI, values greater than .90 were used as an indication of a good model fit. A RMSEA of less than .06 also indicates a good model fit. Standardized path coefficients were used as effect sizes of associations between two variables controlling for other variables in the model. Bootstrapping (Efron & Tibshirani, 1994) was used to address the potential issues of unstable standard error estimation resulted from small samples within groups and non-normal distributions of the variables. SEM was conducted using IBM SPSS AMOS (Chicago, Illinois).

### Results

#### Participants.

There were 638 unique visits to the smoker survey and 1259 unique visits to the nonsmoker survey. Among smokers, 200 were found eligible. Among nonsmokers, 182 nonsusceptible and 190 susceptible were found eligible. Among smokers, 101 were randomized to the imagination arm; among nonsmokers, 92 nonsusceptible and 94 susceptible were randomized to the imagination arm. The demographic and tobacco use profile by arm is presented in Table 1. Overall, smokers were younger than nonsuceptible nonsmokers and there fewer Hispanics among nonsuceptible nonsmokers than susceptibles and smokers. Further, smokers used more tobacco products, followed by susceptible and nonsusceptible nonsmokers.

#### Experiences simulating WTS.

As predicted, smokers reported the most positive thoughts, feelings, and physical sensations related to simulating WTS followed by susceptible nonsmokers and then nonsusceptible nonsmokers (see top of Table 2). This same pattern of effects by smoking status were found for degree of realism and likelihood of simulated event occurring; further, simulating WTS was easiest for smokers and hardest for nonsusceptible nonsmokers. Effort devoted to the simulation did not differ by smoking status.

#### Effects on attitudes and harm perceptions.

We predicted that susceptible nonsmokers and smokers who simulated WTS would report more positive cognitive and affective attitudes and lower perceived harm compared to their counterparts in the control arm; among nonsusceptible nonsmokers, we expected more negative attitudes and higher perceived harm in the simulation than control arm. Analyses are based on 549 observations. Contrary to predictions, for cognitive and affective attitudes (p<.77 and p<.30, respectively) and perceived harm (p <.97) there were no significant interactions. A main effect for arm was found for affective attitudes [p<.03, ω^2^ = .01 (.00, .03)]; participants in the simulation arm reported more positive affective attitudes about WTS than participants in the control arm [M=5.12, SD=2.74, SE=.11, M=4.78, SD=2.61, SE=.10].

#### Effects on immediate desire to smoke.

We predicted an interaction such that susceptible nonsmokers and smokers in the simulation arm would more strongly desire to smoke waterpipe tobacco than their counterparts in the control arm; the opposite pattern was expected for nonsusceptible nonsmokers. Analyses are based on 542 observations. Consistent with predictions, the main effects of smoking status [p<.0001, ω^2^ = .43 (.37, .48)] and study arm [p<.0001, ω^2^ = .43 (.37, .48)] were qualified by their interaction [p<.004, ω^2^ = .02 (.00, .05)]. Whereas desire to smoke did not differ between arms for nonsusceptible nonsmokers (M=1.24, SD=.87, SE=.16 vs. M=1.14, SD=.74, SE=.16, p<.65) and susceptible nonsmokers (M=4.12, SD=1.72, SE=.15 vs. M=3.74, SD=1.66, SE=.16, p<.10), smokers in the imagination arm reported a stronger desire to smoke than those in the control arm (M=4.63, SD=1.73, SE=.15 vs. M=3.50, SD=1.81, SE=.15, p<.0001).

#### Pathways from simulated experiences to desire to smoke.

**Figure 1:**
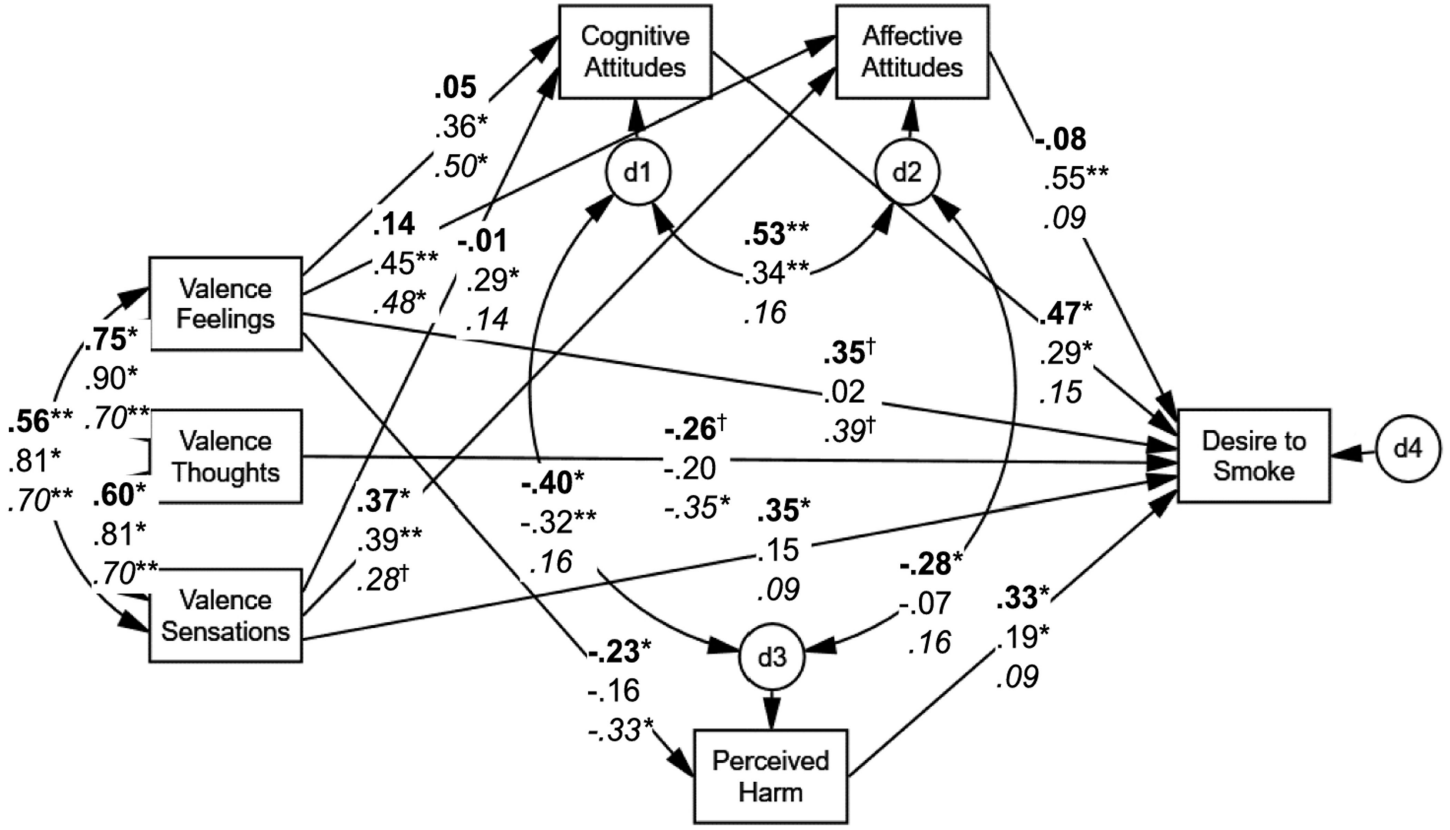
Unconstrainted Mediational Model Predicting Desire to Smoke from Valence of Reactions to Simulation Scenario by Smoking Status (Study 1) Model-fit Indices: χ^2^(12) = 8.68, *p* = .73; GFI = .99, NFI = .99, IFI = 1.00, RFI = .96, CFI = 1.00; RMSEA < .001. All path coefficients are standardized estimates. Values are in **bold** text for smokers (*n* = 97), regular text for susceptible nonsmokers (*n* = 86), and *italicized* text for nonsusceptible nonsmokers (*n* = 86). ^†^*p*< .10. *p< .05. **p< .01.

The multi-group SEM revealed that the unconstrained model (Figure 1) fit the data (χ^2^(12) =8.68, *p* = .73; GFI = .99, NFI = .99, IFI = 1.00, RFI = .96, CFI = 1.00; RMSEA < .001) better than the constrained model with Δχ^2^(22) = 73.59 (*p*< .001), indicating that relations among constructs varied by smoking status. The predicted patterns were supported. Among smokers, greater positive simulated physical sensations were positively and directly associated with a higher immediate desire to smoke (β = 0.35, *p*< .05). Further, more positive simulated feelings were associated with a lower desire to smoke in a mediated pathway: positive simulated feelings were associated with lower perceived harms which, in turn, was associated with lower desire to smoke (β_1_×β_2_ = −0.23×0.33, both *p*s < .05). Among susceptible nonsmokers, none of the simulation reactions were directly associated with desire to smoke. Rather, higher simulated positive feelings and physical sensations were associated with a higher desire to smoke via their relations with more favorable cognitive (β_1_×β_2_ = 0.36×0.29, both *p*s < .05; and β_1_×β_2_ = 0.39×0.29, both *p*s < .05; respectively) and affective (β_1_×β_2_ = 0.45×0.55, both *p*s < .01; and β_1_×β_2_ = 0.39×0.55, both *p*s < .01; respectively) attitudes. Unexpectedly, among nonsusceptible nonsmokers only greater positive thoughts during the simulation were directly associated with a lower desire to smoke (β = −0.35 *p*< .05). For this group, there was no mediated pathway.

## Study 2: Effect of Varying the Valence of Mental Simulation on Desire to Smoke Waterpipe Tobacco

Study 1 provided initial evidence that mental simulations of WTS, especially the valence of simulated experiences, evoked a greater desire to smoke, especially among smokers -- and smokers reported that their simulations closely resembled their smoking experiences. However, experimental evidence is needed that manipulation of valence of simulations motivate desire to smoke waterpipe tobacco. The purpose of Study 2 was to corroborate findings from Study 1 by experimentally *manipulating* valence of simulations (no instruction, positive, negative) and testing whether positive simulations increase the desire to smoke waterpipe tobacco and negative simulations decrease that desire relative to a no simulation control arm. Further, given Study 1 findings that simulating WTS had the most profound effects on smokers followed by susceptible nonsmokers, only these two groups were approached. We predicted that, compared to the no-simulation control arm, participants instructed to simulate positive experiences would report a higher desire to smoke waterpipe tobacco while those instructed to simulate negative experiences would report a lower desire to smoke. No a-priori prediction was made as to how the latter two arms would differ in desire to smoke compared to the no-valence (i.e., unspecified) instruction simulation control arm.

### Methods

#### Participants and online procedures.

Participant recruitment, eligibility, and data quality procedures were the same as in Study 1, except that we used Turkprime to recruit AMT participants (Litman et al., 2017). A $2.00 credit was given to eligible participants who completed the study. Using a between-subjects design, and ran as separate studies by smoking status, eligible susceptible nonsmokers and smokers were randomized with equal probability to one of four experimental arms, described below.

##### No simulation control.

These participants only completed measures.

##### Non-valenced (i.e., unspecified) simulation control.

These participants were asked to simulate WTS without specific instructions of the positivity/negativity (i.e., valence) of the experience. They were instructed to: “Imagine in vivid detail what it would be like for you to smoke waterpipe tobacco (i.e., hookah) in the future. Let your mind roam free and fully immerse yourself in the experience of smoking waterpipe tobacco. This experience may include your surroundings, physical sensations (e.g., taste smell, touch), and thoughts and feelings. Focus your experience while you are smoking waterpipe tobacco.”

##### Positive/negative valanced simulation.

These participants were asked to imagine WTS as a positive or negative experience. They were instructed to: “Imagine in vivid detail what it would be like for you to have a positive/negative experience smoking waterpipe tobacco (i.e., hookah) in the future. Let your mind roam free and fully immerse yourself in the positive/negative experience of smoking waterpipe tobacco. This experience may include your surroundings, physical sensations (e.g., taste smell, touch), and thoughts and feelings that are pleasant/satisfying or unpleasant/unsatisfying to you. Focus only on your positive/negative experience while you are smoking waterpipe tobacco.”

Participants clicked on a button when ready to imagine WTS. Upon clicking the button, they were informed they had 20 seconds to imagine WTS per the initial instructions (e.g., “You have 20 seconds to imagine this negative experience of smoking waterpipe tobacco”). Consistent with prior studies of episodic future thinking (e.g., Addis et al., 2007), simulation time was 20 seconds to allow participants sufficient time to generate detailed scenarios. After 20 seconds, they were taken to a page instructing them to describe their positive, negative, or unspecified valenced simulated experience while smoking waterpipe tobacco. The survey was programmed such that participants in the experimental arms were not allowed to continue further unless they entered text (at least three characters) describing their experiences. After detailing their account, they and the no simulation control arm participants completed the measures below.

#### Measures

Participants in the three simulation arms first completed the same measures as in Study 1 capturing reactions to their experience with valence of simulated thoughts, feelings and physical sensations serving as the main manipulation checks. All participants reported on their attitudes using the same 10-item scale as in Study 1. Perceived harm was assessed by the mean response to two questions, one about chance of getting a serious smoking-related disease as in Study 1, and by: “Your “gut feeling” tells you that you are hurting your health when you smoke waterpipe tobacco?” (1=Strongly disagree to 7=Strongly agree); the average Pearson correlation across arms and smoking status was .53 (range .37 to .66). All participants were asked about their desire to smoke waterpipe tobacco right now (1=Not at all strong to 7=Very strong), and as an intentionmeasure, their likelihood of WTS during the next month (1=No chance to 7=Certain to happen).

#### Analytical methods

Mean reactions to the simulated scenarios were analyzed as a between-subjects design via 2 (smoker/susceptible nonsmoker) x 3 (unspecified-/positive-/negative-valenced simulation) ANOVAs. Mean reactions to cognitive/affective attitudes, perceived harm, and desire to smoke were analyzed as a between-subjects designs via 2 (smoker/susceptible nonsmoker) x 4 (nosimulation /unspecified-/positive-/negative-valenced simulation condition) ANOVAs. Analyses were two-tailed using p<.05 as statistical significance and conducted with SAS Version 9.4 (Cary, North Carolina). Demographic variables did not differ by study arm. Smoking profile did not vary by arm except that more participants used regular pipe in the no simulation control than the other arms.

Moderated mediational analyses were conducted using multi-group SEM modelling the effects of smoking status and simulation arm on desire to smoke using cognitive/affective attitudes and perceived harm as mediators and smoking status as a moderator. For these 2 × 4 analyses, we contrasted effects of each simulation condition against the no simulation arm. We focused the moderated mediational analyses on desire to smoke for consistency with Study 1. As in Study 1, we tested fit of these data in constrained and unconstrained models using bootstrapping and the same fit indices. SEM was undertaken using IBM SPSS AMOS (Chicago, Illinois).

### Results

#### Participants.

There were 1507 unique visits to the smoker survey and 6264 unique visits to the susceptible nonsmoker survey. Among smokers, 234 were found eligible. Among susceptible nonsmokers, 241 were found eligible. Overall, 113 to 129 participants were randomized to each of the four arms, with 56 to 64 smokers and 56 to 65 susceptible nonsmokers per each of the four study arms. The demographic and tobacco use profile by arm is presented in Table 1 on the right. Smokers were younger than susceptible nonsmokers. Further, smokers were more likely to be employed full time.

#### Experiences simulating WTS.

It was expected that participants in the positive-valence simulation arm would report the most positive simulated thoughts, feelings, and physical sensations, followed by those in the control arm and then the negative-valence arm. As shown at the top of Table 3, these arm main effects were supported; in general, smokers reported greater positive reactions for these outcomes than susceptible nonsmokers. Further, significant arm by smoker interactions indicated that unlike susceptible nonsmokers, smokers’ thoughts, feelings, and physical sensations did not differ between being instructed to simulate a positive experience versus when given no simulation instructions.

There were no significant main effects of simulation arm for realism of simulated event, ease, or effort invested simulating the scenario. Simulation arm did interact with smoking status for realism of and likelihood of experiencing the simulated scenario. Compared to smokers, susceptible nonsmokers reported their scenarios as less realistic overall (M=4.46, SD= 1.70, SE=.11 vs. M=5.81, SD=1.30, SE=11); they viewed the difference in the realism of positive and negative events as less than smokers (M=.36, vs. .66). Further, while susceptible nonsmokers viewed the likelihood of experiencing the scenario as similar across instructions, smokers viewed negative scenarios as the least likely to occur relative to the other conditions – which did not differ. Susceptible nonsmokers had more difficulty imagining WTS than smokers (M=3.59, SE=.12 vs. M=2.22, SE=.12) and they put more effort into imagining WTS (M=5.57, SE=.12 vs. M=5.13, SE=.12).

In sum, the manipulations worked in the hypothesized directions. For smokers, however, the no-valence control and positive-valence simulation arms often did not differ yet varied significantly from the negative-valence simulation arm on outcomes other than ease and effort put into simulating scenario.

#### Effects on attitudes and perceived harm.

As shown in Table 4, participants reported significantly more favorable cognitive attitudes in the positive-valence simulation arm compared to any other condition, which did not differ from each other. Participants reported the most negative affective attitudes in the negative-valence simulation arm and reported the most favorable affective attitudes in the positive-valence simulation arm; the two control arms did not differ. On average, smokers had more positive attitudes than susceptible nonsmokers. There were no significant interactions. Participants in the positive-valence simulation arm viewed harms of WTS as lower than the remaining three arms, which did not differ between themselves. Smokers viewed the harms of WTS as lower than susceptible nonsmokers. There were no significant interactions.

#### Effects on immediate desire and future likelihood of WTS.

Provision of no-valence or positive-valence instructions produced the highest immediate desire to smoke waterpipe tobacco compared to the other conditions. With respect to future smoking, participants in the negative-valence simulation arm reported a lower likelihood of engaging in WTS during the next month compared to the positive-valanced arm; however, neither of these two arms differed from both control arms. Smokers reported a stronger desire to and higher likelihood of smoking waterpipe in the next month than susceptible nonsmokers. There were no significant interactions.

#### Moderated mediational analyses on desire to smoke.

**Figure 2:**
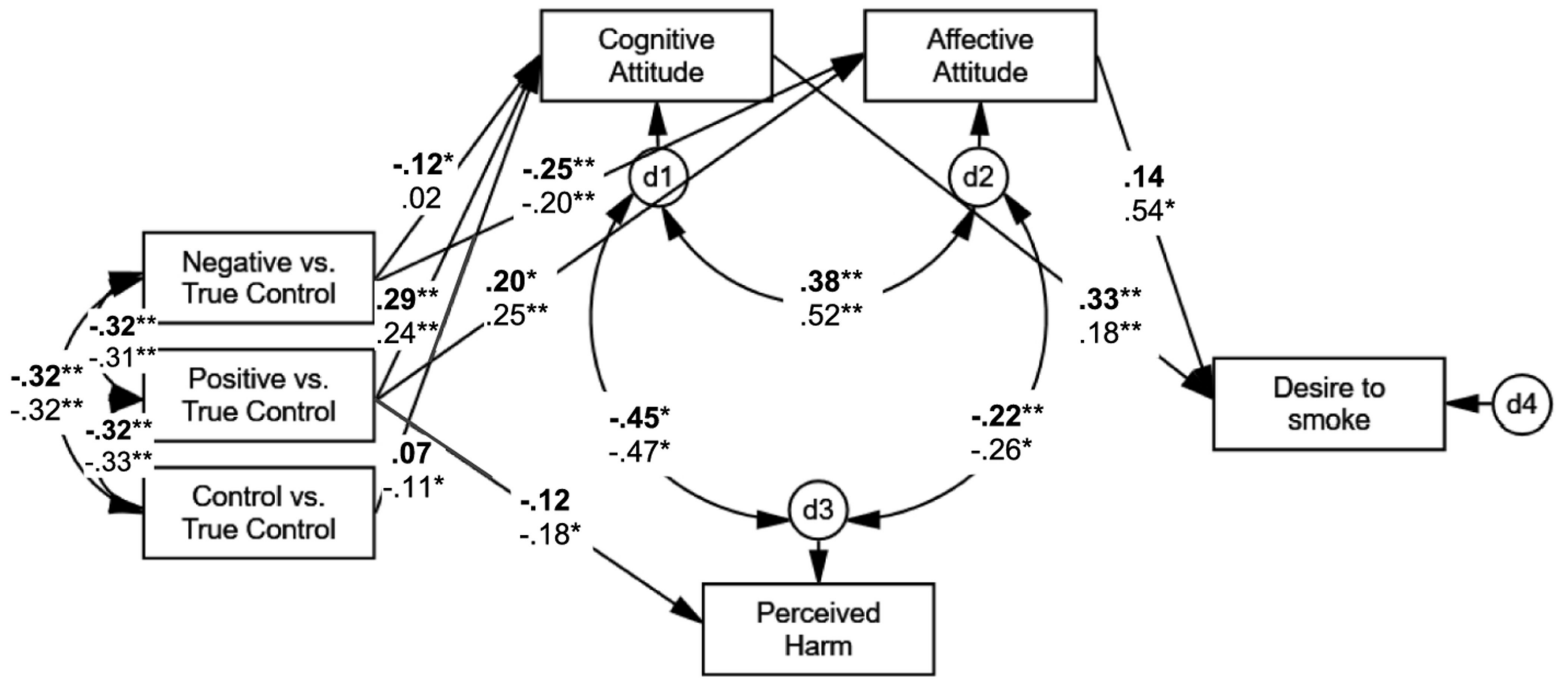
Unconstrainted Mediational Model Predicting Desire to Smoke Comparing Three Simulation Arms to Control by Smoking Status (Study 2) Model-fit Indices: χ^2^(14) = 23.60, *p* = .051; GFI = .99, NFI = .97, IFI = .99, RFI = .91, CFI = .99; RMSEA = .04. All path coefficients are standardized estimates. Values are in **bold** text are for smokers (*n* = 234); values in regular text are for susceptible nonsmokers (*n* = 241). True control is the no simulation control arm. *p< .05. **p< .01.

The multi-group SEM results demonstrated that the unconstrained SEM (Figure 2) fit the data (χ^2^(14) = 23.60, *p* = .051; GFI = .99, NFI = .97, IFI = .99, RFI = .91, CFI = .99; RMSEA = .04.) better than the constrained model with Δχ^2^(8) = 30.64 (*p*< .001), indicating that relations among constructs varied by smoking status. Among smokers, there was only one complete mediational pathway that resulted in a higher desire to smoke. Smokers randomized to the positive-valenced simulation arm, compared to control arm, had more favorable cognitive attitudes that resulted in a higher desire to smoke (β_1_×β_2_ = 0.29×0.33, both *p*s < .01). While smokers randomized to this arm did report more positive affective attitudes (β = 0.20, *p*< .05), the latter failed to be significantly related to desire to smoke (β = 0.14, *n.s.*). Further, there was only one complete mediational pathway that resulted in a lower desire to smoke. Smokers randomized to the negative-valenced simulation arm, compared to the control arm, reported less favorable cognitive attitudes, which in turn was related positively with a higher desire to smoke – total path was negative (β_1_×β_2_ = −0.12×0.33, *p*_1_< .05 and *p*_2_< .01).

Among susceptible nonsmokers, there were two complete pathways that resulted in a higher desire to smoke. Those randomized to the positive-valenced simulation arm reported more favorable cognitive and affective attitudes compared to the control arm; these attitudes, in turn, were related to a greater desire to smoke (β_1_×β_2_ = 0.24×0.18, both *p*s < .01; and β_1_×β_2_ = 0.25×0.18, both *p*s < .01; respectively). Further, there was one complete mediational pathway that resulted in a lower desire to smoke. Those randomized to the no-valence simulation control arm reported less favorable cognitive attitudes compared to the control arm, which in turn was positively related to a higher desire to smoke – total negative path (β_1_×β_2_ = −0.11×0.18, *p*_1_< .05 and *p*_2_< .01).

## Discussion

In two studies, we showed that engaging in mental simulations of future WTS influenced the immediate desire to smoke compared to no episodic future mental simulations. Study 1 provided initial insights about smokers’ and nonsmokers’ experiences of mentally simulating future WTS. According to the constructive episodic simulation hypothesis (Schacter & Addis, 2007), smokers’ encoded memories of past experiences of WTS should more strongly influence the valence of simulated future thoughts, feelings, and physical sensations and result in more easily generated and realistic scenarios when simulating future episodes, compared to nonsmokers. Findings were consistent with these predictions whereby smokers had the most favorable experiences and found the simulated scenarios realistic, more likely to resemble a future experience, and easier to produce. Moreover, smokers who simulated WTS (versus those who did not) expressed a stronger desire to smoke; there was a similar (but non-significant) pattern for susceptible nonsmokers. Among nonsusceptible nonsmokers, the mental simulation had no significant effect on desire to smoke. Modeling demonstrated different pathways through which the mental simulation affected desire to smoke by smoking status. For both smokers and susceptible nonsmokers, there were more direct (e.g., physical sensation for smokers) or fully mediated indirect paths linking reactions with attitudes or perceived harms, relative to nonsusceptible nonsmokers. That is, for smokers and susceptible nonsmokers, mental simulations appear to have more routes through which they can influence desire to smoke WTS than for nonsusceptible nonsmokers. Nonsusceptible nonsmokers lacked full mediational pathways linking valence of simulated reactions with desire to smoke.

The purpose of Study 2 was to corroborate and extend Study 1 findings by experimentally manipulating valence of simulations and testing whether positive simulations increase the desire to smoke waterpipe tobacco and negative simulations decrease that desire relative to a no simulation control. Consistent with Study 1, across manipulations smokers had more positively-valenced thoughts, feelings, and physical sensations, found it easier and less effortful to generate simulations, and simulations were judged as more realistic and likely to be experienced than susceptible nonsmokers. Valence of thoughts, feelings, and physical reactions were similar when smokers, but not susceptible nonsmokers, were induced to simulate positive experiences or when provided with no specific valenced instructions.

Varying the valence of mental simulations helped clarify why smokers asked to simulate WTS in Study 1 reported a greater desire to smoke than those in the control arm. Findings suggest that when asked to simulate future WTS, smokers in Study 1 likely defaulted to positive thoughts, feelings, and/or physical sensations akin to giving them instructions to simulate positive scenarios, resulting in similar effects on desire to smoke. Providing no instructions about valence or instructions to produce positive simulations resulted in similar increases in desire to smoke, especially compared to participants who were not asked to produce simulations.

Study 2 SEM analyses provided further evidence of mediation effects of simulation conditions on desire to smoke relative to a no simulation control. Engaging in positive simulations heightened desire to smoke for smokers via favorable cognitions and for susceptible nonsmokers via both favorable cognitive and affective attitudes. Conversely, negative simulations lowered desire to smoke for smokers via the reduction of favorable cognitive attitudes and for susceptible nonsmokers via more negative affective attitudes. Among susceptible nonsmokers, allowing them to freely simulate WTS with unspecified valence instructions reduced the immediate desire to smoke by decreasing favorable cognitive attitudes.

Study 2 also examined the likelihood of engaging in WTS one-month into the future. Here we found that negative simulations reduced the likelihood of future smoking compared to the positive simulation arm. Thus, whereas the immediate desire to smoke waterpipe was influenced by positive simulations, negative simulations played a significant role both in reducing the perceived likelihood of smoking in the next month and in diminishing the desire to smoke right now. These findings suggest that negatively-valenced simulations could be harnessed as a preventive intervention and used to counter marketing that exploits the impact of positive simulations on WTS (Palmedo et al., 2017). It will be important to test potential strategies (e.g., public health messaging, simulations embedded into behavioral interventions) that can be leveraged to induce negative simulations to prevent WTS and encourage cessation in future studies.

Our findings have potential theoretical and practical implications concerning WTS. First, mental simulations have not been researched as a mechanism that influences WTS and should be integrated into the WTS literature. Simulations affected how smokers and susceptible nonsmokers evaluated WTS immediately thereafter; they mattered. Second, simulations can act as important *precursors* to cognitions and affective attitudes that influence desire and intentions to engage in WTS. Indeed, simulations influenced cognitive and affective attitudes as mediators, with weak or no effects on perceived harms. Suggested by our findings, marketers may promote positive simulations of the experience of smoking waterpipe tobacco, influencing susceptible nonsmokers’ experimentation with WTS through both cognitive and affective mechanisms while affecting smokers through cognitive appeals, directing their thinking about the WTS experience (Papies et al., 2017). Third, our findings show that we can explicitly influence valence of simulations. Negative simulations need to be reinforced and positive simulations interrupted; for example, use of health warnings or making explicit the motives and misleading claims of marketers (Sutfin et al., 2021) may achieve both these ends. Fourth, the study of mental simulation can inform policy to curb WTS. Ads promoting WTS likely trigger positive simulations that increase product use. Research is needed as to how various ad attributes (e.g., size, color, placement, descriptors of WTS) affect content and valence of simulations and ensuing thoughts, feelings, and behaviors. Policy-makers can consider banning ad attributes that produce positive simulations that result in WTS – akin to disallowing the term “light” cigarettes.

This research contributes to the larger literature linking episodic future mental simulations with behavior modification. For example, a recent review of 123 studies by Cole et al. (Cole et al., 2020) observed a significant, albeit modest, effect of mental simulations on behavior change (g=.49). Although the review included a wide variety of behaviors, many studies were health-related such as reduction in alcohol consumption (Hagger et al., 2011; 2012) and calorie intake (Johannessen et al., 2012). To our knowledge, the results reported here constitute the first evidence that episodic future simulation may be an effective strategy to curb future WTS behavior, and thus extends the scope of simulation research to an important health-risk behavior.

There are several limitations to our findings. First, it is unclear how well the findings generalize to populations beyond the current sample. Convenience samples like the one used here represent an important first step to test key hypotheses but we acknowledge that the hypotheses tested here should be examined in more representative samples. Second, we did not assess the durability of effects or, crucially, the uptake or cessation of WTS. It is possible that mental simulation may affect instrumental behaviors such as telling oneself to stop while smoking waterpipe (i.e., self-talk) yet require repeated and consistent practice to modify smoking behavior. Third, while mental simulations in general (Study 1) and the valence of the mental simulations (Study 2) were manipulated, instructions pertaining to what exactly to simulate (e.g., type of outcome, context, etc.) were not provided. It is likely that different simulations (e.g., smoking alone or with friends, a specific type of negative or positive outcome) could produce different effects. Testing guided simulations will be important in future research.

Notwithstanding these limitations, these findings reveal that mental simulations can influence key beliefs and the desire to smoke in smokers and susceptible nonsmokers in a new health domain, WTS. Mental simulations are highly malleable, often used in planning for the future, and can change health behaviors (Cole et al., 2020; Conroy & Hagger, 2018). Given the paucity of novel strategies to prevent and promote cessation of WTS in young adults, interventions that use and build on mental simulations merit further scrutiny.

## Figures and Tables

**Table 1:** Demographics and Tobacco Use Profiles in Total Sample and by Smoking Status in Study 1 and Study 2

	Study 1					Study 2			
Outcome	Total Sample	Nonsmoker		Smokers	p <	Total Sample	Susceptible Nonsmoker	Smokers	p<
		Nonsusceptible	Susceptible						
Mean age (SD)	26.2 (2.90)	26.4_ab_ (2.81)	26.6_a_ (2.86)	25.8_b_ (2.97)	.02	25.0 (3.20)	24.2 (3.39)	25.6 (2.86)	.0001
Males (N, %)	339, 61.8	101, 29.8	114, 33.6	124, 36.6	.42	220, 46.3	94, 42.7	126, 57.3	.002
Race (N, %)									
White	421, 80.5	140 33.2	136 32.3	145 34.4	.53	344, 73.7	176, 51.2	168, 48.4	.08
African American	60, 11.5	17 28.3	22 36.7	21 35.0		53, 11.4	19, 35.8	34, 64.2	
Asian	42, 8.0	13 31.0	10 23.8	19 45.2		37, 7.9	23, 62.2	14, 37.8	
Other	25, 4.6	5 20.0	11 44.0	9 36.0		33, 7.1	15, 45.5	18, 54.6	
Hispanic (N, %)	59, 10.8	10, 17.0_a_	21, 35.6_b_	28, 47.5_b_	.03	57, 12.2	28, 49.1	29, 50.9	.91
Educations (N,%)									
High school or less	80, 14.6	26, 32.5	18, 22.5	36, 45.6	.13	57, 12.2	33, 57.9	24, 42.1	.44
Some college	208, 37.9	59, 28.4	75, 36.1	74, 36.6		186, 39.8	91, 48.9	95, 51.1	
College graduate/Post	261, 47.5	90, 34.5	86, 33.0	85, 32.6		224, 48.0	109, 48.7	115, 51.3	
Current student (N, %)	98, 18.0	26, 26.5	33, 33.7	39, 39.7	.43	162, 34.7	84, 51.5	78, 48.2	.54
Employment (N, %)									
Full time	381, 69.4	117, 30.7	120, 31.5	144, 37.8	.51	265, 56.8	97, 36.6	168, 63.4	.0001
Part time	90, 16.4	29, 32.2	33, 36.7	28, 31.1		120, 25.7	77, 64.2	43, 35.8	
Not employed	78, 14.2	29, 37.2	26, 33.3	23, 29.5		82, 17.6	59, 72.0	23, 28.0	
Tobacco products last 30 days (N, %)									
Cigarettes	158, 27.6	15, 9.5_a_	39, 24.7_b_	104, 65.8_c_	.0001	90, 19.0	10 11.1	80, 89.9	.0001
Large cigars	33, 5.8	1, 3.0_a_	7, 21.2_b_	25, 75.8_b_	.0001	22, 4.6	6, 27.3	16, 72.7	.03
Little cigars	46, 8.0	3, 6.5_a_	6, 13.0_a_	37, 80.4_b_	.0001	43, 9.0	6, 14.0	37, 86.0	.0001
Electronic cigarette	75, 13.1	3, 4.0_a_	15, 20.0_b_	57, 76.0_b_	.0001	72, 15.2	17, 23.6	55, 76.4	.0001
Regular pipe	35, 6.1	1, 2.9_a_	5, 14.3_a_	29, 82.9_b_	.0001	30, 6.3	2. 6.7	28, 93.3	.0001
Smokeless	18, 3.2	2, 11.1_a_	3, 16.7_a_	13. 72.2_b_	.004	13, 2.5	1, 8.3	11, 91.7	.0001

Note. Means and percentages with different lettered subscripts differ at p≤.05. The total number of observations for some variables may not add up to the sample sizes for study 1 (N=572) and Study 2 (N=475) due to missing data. Percentage may not add to 100% due to rounding. The “other” category for race includes Native Hawaiian or other Pacific Islander, American Indian or Alaska Native, Middle Easterners and participants who reported mixed race.

**Table 2:** Reported Experience of Simulating Waterpipe Tobacco Smoking by Smoking Status (Study 1)

Outcome	Smoking Status			Main effect for smoking status p <, effect size, ω^2^, (95% CI)
	Non-susceptible nonsmoker (n=88)	Susceptible Nonsmoker (n=87)	Smoker (n=97)	
Valence of simulated thoughts	2.01_a_ (1.33, .15)	4.76_b_ (1.33, .15)	5.88_c_ (1.34, .14)	.0001 .57 (.50, .63)
Valence of simulated feelings	2.12_a_ (1.44, .15)	4.80_b_ (1.46, .15)	5.91_c_ (1.22, .14)	.0001 .57 (.50, .63)
Valence of simulated physical sensations	2.14_a_ (1.38, .14)	5.01_b_ (1.34, .14)	5.86_c_ (1.14, .13)	.0001 .60 (.54, .66)
Realism of simulated experience	3.77_a_ (1.90, 1.6)	5.20_b_ (1.26, .16)	6.00_c_ (1.13, .16)	.0001 .28 (.20, .37)
Likelihood of experiencing WTS as imagined	1.70_a_ (1.35, .14)	4.84_b_ (1.38, .14)	5.85_c_ (1.30, .14)	.0001 .63 (.60, .68)
Ease of simulating future WTS	4.60_a_, (1.94, .18)	3.50_b_ (1.73, .19)	2.25_c_ (1.55, .18)	.0001 .23 (0.15, .32)
Effort devoted to simulating WTS	5.59 (1.48, .17)	5.41 (1.51, .17)	5.28 (1.70, .16)	.41 .00 (.00, .03)

Note. Numbers in parenthesis represent the standard deviation followed by the standard error of the mean. Higher means represent more positive valence of thoughts, feelings and physical sensations as well as greater realism, likelihood and effort. Contrast of means with different lettered subscripts differ by p<.05.

**Table 3:** Reported Experience of Simulating Waterpipe Tobacco Smoking by Imagined Study Arm and Smoking Status (Study 2)

Outcomes	Susceptible nonsmokers			Smokers			p <, effect size, ω^2^, (95% CI)		
	Control (n=61)	Positive (n=58)	Negative (n=56)	Control (n=56)	Positive (n=57)	Negative (n=57)	Imagined valence	Smoking status	Interaction
Valence of simulated thoughts	4.03_a_ (1.90, .17)	5.36_b_ (1.25, .18)	2.18_c_ (0.86, .18)	6.09_d_ (1.07, .18)	6.26_d_ (1.22, .18)	2.65_c_ (1.44, .18)	.0001 .54(0.47, .60)	.0001 .15 (.09, .22)	.0001 .05 (.02, .11)
Valence of simulated feelings	4.11_a_ (1.78, .17)	5.38_b_ (1.09, .17)	2.41_c_ (1.09, .18)	6.12_d_ (1.06, .18)	6.23_d_ (1.16, .17)	2.61_c_ (1.48, .17)	.0001 .53(0.47, .59)	.0001 .13 (.07, .20)	.0001 .07 (.03, .13)
Valence of simulated physical sensations	4.31_a_ (1.78, .16)	5.26_b_ (1.12, .17)	2.11_c_ (0.95, .17)	6.11_d_ (0.93, .17)	6.21_d_ (0.92, .17)	2.86_e_ (1.61, .17)	.0001 .55(0.48, .60)	.0001 .17 (.11, .24)	.005 .02 (.00, .07)
Realism of simulated experience	4.57_a_ (1.84,.19)	4.22_a_ (1.61, .20)	4.57_a_ (1.65, .20)	5.71_b_ (1.23, .20)	6.19_b_ (1.12, .20)	5.53_b_ (1.44, .20)	.73 .00 (.00, .02)	.0001 .17 (.10, .24)	.03 .02 (.00, .06)
How likely to experience WTS as simulated	3.00_a_ (1.72, .19)	3.31_a_ (1.51, .19)	2.84_a_ (1.51, .19)	5.98_b_ (1.17, .19)	6.16_b_ (1.10, .19)	4.56_c_ (1.66, .19)	.0001 .05 (.01, .10)	.0001 .40 (.32, .47)	.002 .02 (.00, .05)
Ease of simulated WTS	3.48_a_ (1.81, .20)	3.55_a_ (1.48, .21)	3.73_a_ (1.70, .21)	2.32_b_ (1.62, .21)	1.93_b_ (1.46, .21)	2.40_b_ (1.51, .21)	.31 .00 (.00, .03)	.0001 .15 (.09, .22)	.54 .00 (.00, .02)
Effort put into simulating WTS	5.72_a_ (1.14, .20)	5.41_b_ (1.40. 20)	5.57_b_ (1.22, .21)	5.05_c_ (1.78, .21)	5.10_c_ (1.99, .21)	5.23_c_ (1.65, .21)	.76 .00 (.00, .02)	.01 .02 (.00, .06)	.63 .00 (.00, .02)

Note. Numbers in parenthesis represent the standard deviation followed by the standard error of the mean. Higher means represent more positive valence of thoughts, feelings and physical sensations as well as greater realism, likelihood and effort. Contrast of means with different lettered subscripts differ by p<.05.

**Table 4:** Effects of Attitudes, Risk Perceptions and Smoking Outcomes by Study Arm and Smoking Status

Outcomes	Simulation Arm				Arm main effect, p-value, effect size, ω^2^, (95% CI)	Smoking status		Smoking status main effect p-value, effect size, ω^2^, (95% CI)
	No Simulation (n=129)	Non-valenced Simulation (n=117)	Positive Valence (n=115)	Negative Valence (n=113)		Smoker (n=234)	Susceptible Nonsmoker (241)	
Cognitive attitudes	3.76_a_ (1.65, .12)	3.61_a_ (1.80, .13)	4.62_b_ (1.82, .13)	3.48_a_ (1.42, .13)	.0001 .08 (.04, .13)	4.78 (1.57, .09)	2.97, (1.37, .09)	.0001 .29 (.23, .35)
Affective attitudes	5.50_a_ (1.65, .12)	5.66_a_ (2.47, .13)	6.53_b_ (1.97, .13)	4.62_c_ (2.06, .13)	.0001 .13 (.08, .09)	6.94 (1.74, .09)	4.25, (1.91, .09)	.0001 .38 (.32, .44)
Harm perceptions	4.59_a_ (1.40, .10)	4.88_a_ (1.36, .10)	4.39_b_ (1.36. .10)	4.84_a_ (1.23. .10)	.003 .02 (.00, .06)	3.91 (1.22, .07)	5.43 (1.01, .07	.0001 .32 (.25, .38)
Desire to smoke right now	2.96_a_ (1.79, .14)	3.34_b_ (1.96, .14)	3.74_b_ (1.95, .14)	2.87_a_ (1.74, .14)	.0001 .04 (.01, .09)	4.26 (1.59, .10)	2.21 (1.58, .10)	.0001 .30 (.24, .37)
Likelihood of WTS next month	3.91_a_ (1.68, .13)	3.80_a_ (1.80, ,14)	4.21_ab_ (1.68, .14)	3.54_ac_ (1.68, .14)	.009 .02 (.00, .05)	4.67 (1.40, .10)	3.10 (1.63 .10)	.0001 .53 (.48, .58)

Note. Numbers are means, standard deviations and standard error of the means. Higher mean scores reflect more positive cognitive and affective attitudes, greater perception harm, a stronger immediate desire to smoke, and higher likelihood in engaging in WTS during the next month. Means with different lettered subscripts differ by p<.05. There were no arm by smoking status interactions.
